# Aromadendrin Ameliorates Airway Inflammation in Experimental Mice with Chronic Obstructive Pulmonary Disease

**DOI:** 10.4014/jmb.2408.08022

**Published:** 2024-11-19

**Authors:** Jinseon Choi, Seok Han Yun, Hyueyun Kim, Juhyun Lee, Seong-Man Kim, Mi-Hyeong Park, Hee Jae Lee, Wanjoo Chun, Sang-Bae Han, Kyung-Seop Ahn, Jae-Won Lee

**Affiliations:** 1 Natural Medicine Research Center, Korea Research Institute of Bioscience and Biotechnology, Cheongju 28116, Republic of Korea; 2 College of Pharmacy, Chungbuk National University, Cheongju 28160, Republic of Korea; 3 O_2_MEDi Inc. 50 UNIST-gil, Eonyang-eup, Ulju-gun, Ulsan 44919, Republic of Korea; 4 Office of Surveillance for Narcotics Abuse, Ministry of Food and Drug Safety, Osong Health Technology Administration Complex, Cheongju 28159, Republic of Korea; 5 Department of Pharmacology, College of Medicine, Kangwon National University, Chuncheon 24341, Republic of Korea; 6 Department of Biotechnology, University of Science and Technology (UST), Daejeon 34113, Republic of Korea

**Keywords:** COPD, aromadendrin, cytokines, NF-κB, NLRP3 inflammasome

## Abstract

Aromadendrin (ARO) is an active plant compound that exerts anti-inflammatory effects. However, its ameliorative effects on chronic obstructive pulmonary disease (COPD) remain unclear. Therefore, we investigated the inhibitory effects of ARO on bronchial inflammation using an experimental model of COPD. In vivo analysis confirmed a notable increase in the number of neutrophils/macrophages and the formation of reactive oxygen species (ROS), myeloperoxidase (MPO), interleukin (IL)-6/IL-1β, and monocyte chemoattractant protein (MCP)-1 in the bronchoalveolar lavage (BAL) fluid of COPD mice, which was attenuated by oral gavage of ARO. In addition, hematoxylin and eosin staining showed a notable cell influx in the lungs of the COPD group, which was ameliorated by ARO. Western blotting revealed that ARO decreased the upregulation of neutrophil elastase expression in the lungs of the COPD group. Furthermore, periodic acid–Schiff staining showed that increased mucus formation in the lungs of the COPD group was downregulated by ARO. ARO also blocked CREB activation in the lungs of COPD mice. This in vivo, anti-inflammatory effect of ARO was accompanied by its modulatory effect on the activation of the MAPK/NF-κB/NLRP3 inflammasome. In summary, our study demonstrated that ARO has protective effects on bronchial inflammation by attenuating immune cell accumulation, toxic molecule/cytokine/chemokine formation, and MAPK/NF-κB/NLRP3 inflammasome activation, suggesting the potential development of ARO as an adjuvant for the prevention and treatment of COPD.

## Introduction

Chronic obstructive pulmonary disease (COPD) is a serious respiratory ailment resulting in increasing mortality worldwide [1]. Meanwhile, the need for drugs or supplements to treat COPD has become urgent. Smoking and bacterial infection promote the development of COPD by inducing immune cell activation and bronchial inflammation [2]. In COPD, neutrophils induce lung damage by forming reactive oxygen species (ROS), myeloperoxidase (MPO), and elastase [3-5]. Elevated levels of cytokines and chemokines, such as interleukin (IL)-6 [6], IL-1β [7], and monocyte chemoattractant protein (MCP)-1 [8] are observed in patients with COPD. Macrophages produce IL-6/IL-1β/MCP-1 and influence neutrophil influx, promoting bronchial inflammation in COPD [9]. Mucus accumulation limits airflow and affects pulmonary function in COPD [10].

Activation of the mitogen-activated protein kinase (MAPK)/nuclear factor kappa B (NF-κB)/NOD-like receptor protein 3 (NLRP3) inflammasome promotes bronchial inflammation [11-13]. Cigarette smoke extract (CSE) induces the proinflammatory M1 phenotype in alveolar macrophages by activating JNK MAPK [14]. p38 MAPK activation in the lungs of patients with COPD is associated with the development of bronchial inflammation [15, 16]. In vitro and in vivo models of COPD have shown activation of ERK MAPK [17-19]. NF-κB activation is a critical event in COPD development and has been confirmed in the lungs of cigarette smoke (CS)/lipopolysaccharide (LPS)-induced COPD animal models [20]. Activation of the NLRP3 inflammasome promotes the expression of IL-1β and is closely associated with the progression of pulmonary inflammation in COPD [21].

The intranasal administration of LPS accelerated CS-induced bronchial inflammation, similar to that observed in patients with COPD, by promoting neutrophil/macrophage accumulation, ROS/cytokine/chemokine formation, and MAPK/NF-κB activation in experimental COPD mice [18, 22].

Phenolic compounds exhibit various biological effects, including anti-inflammatory activity, according to cumulative in vitro and in vivo studies [18, 23, 24]. A flavanonol, aromadendrin (ARO), also known as dihydrokaempferol, is present in the pulp of *Euterpe oleracea* Martitus [25], the rhizome of *Smilax glabra* [26], *Bauhinia championii* (Benth.) Benth. [27], and the leaves of *Olea europaea* L. [28]. A recent in vitro study demonstrated the anti-inflammatory effects of ARO in keratinocytes [28]. Previously, ARO ameliorated LPS-induced inflammatory response in RAW 264.7 macrophages by downregulating the expression of iNOS/COX-2 and the nuclear translocation of NF-κB [29]. Recently, Park *et al*. confirmed the anti-asthmatic effect of ARO in mice with ovalbumin (OVA)-induced allergic asthma via the inhibition of Th2 cytokines and NF-κB activation [30]. However, as the ameliorative effects of ARO on bronchial inflammation remain unexplored in COPD, we therefore examined these effects using an in vivo model of COPD.

## Materials and Methods

### Reagents

Aromadendrin (ARO) was purchased from the Natural Products Research & Development Enterprise (ChemFaces, China).

### Experimental Mouse Model of COPD

Six-week-old male C57BL/6 mice were purchased from Koatech Co. Ltd. (Republic of Korea). The procedures for animal experiments were approved by the IACUC of KRIBB (KRIBB-AEC-23122).

To establish bronchial inflammation, similar to that observed in COPD, mice were exposed to CS and LPS as previously described [11]. Briefly, the mice were exposed to CS for 50 min/day (seven cigarettes/day) for 7 days using a smoking machine (SciTech Korea, Inc., Republic of Korea). LPS was intranasally injected into the mice on day 6 (5 μg in 40 μl/mouse). Oral gavage (o.g.) of aromadendrin (ARO) and roflumilast (ROF) were administered for 7 consecutive days.

Five experimental groups (*n* = 6 per group) were established as follows: normal control (NC) [mice received normal saline], COPD [mice exposed to CS/intranasal LPS], ROF [COPD + 5 mg/kg ROF], ARO 5 [COPD + 5 mg/kg ARO], and ARO 10 [COPD + 10 mg/kg ARO].

### Analysis of Immune Cells and Molecules in Bronchoalveolar Lavage (BAL) Fluid

To count immune cells in the BAL fluid, the mice were anesthetized with a mixture of Zoletil (30 mg/kg) and xylazine (5 mg/kg) [31]. Cell morphology was distinguished by Diff-Quik staining and cell numbers were measured using a light microscope (400 × magnification).

The level of ROS in BAL fluid was estimated using 2',7'-dichlorodihydrofluorescein diacetate (DCF-DA) based on previous protocols [17]. The levels of IL-6, IL-1β, and MCP-1 in BAL fluid supernatant were measured using specific ELISA kits.

### Western Blotting Analysis

The harvesting of lung tissue lysate with lysis buffer and protein quantification with BCA assay were performed based on a previous study [31] to detect the expression levels of neutrophil elastase (NE) and phosphorylated (p)-CREB/p-JNK/p-p38/p-ERK/p-p65/p-IκBα/NLRP3/ASC/Caspase-1. Each sample was then loaded onto an SDS-PAGE gel and transferred onto a PVDF membrane. Subsequently, membranes were incubated in blocking reagent (1× TBST with 5% skim milk) and primary antibodies (Table 1). The membranes were washed with 1× TBST four times prior to incubation with the corresponding secondary antibodies. Finally, the membranes were exposed to an ECL solution to visualize the bands.

### Histological Analysis

Histological changes in the lungs of mice were determined using hematoxylin and eosin (H&E) and PAS staining. For each staining, the lung tissues dissected from the experimental mice were washed in ice-cold PBS, fixed in 10% formalin solution, and embedded in paraffin. Subsequently, lung tissue sections were stained with H&E and PAS [32].

### Statistical Analysis

Values are expressed as the mean ± SD. One-way analysis of variance (ANOVA) followed by Tukey’s multiple comparison test was performed to analyze differences between groups. Data were analyzed using SPSS software (version 20.0; IBM Corp.). *p* < 0.05 was considered statistically significant.

## Results

### ARO Reduced the Influx of Immune Cells in Mice with COPD

**Fig. 1:**
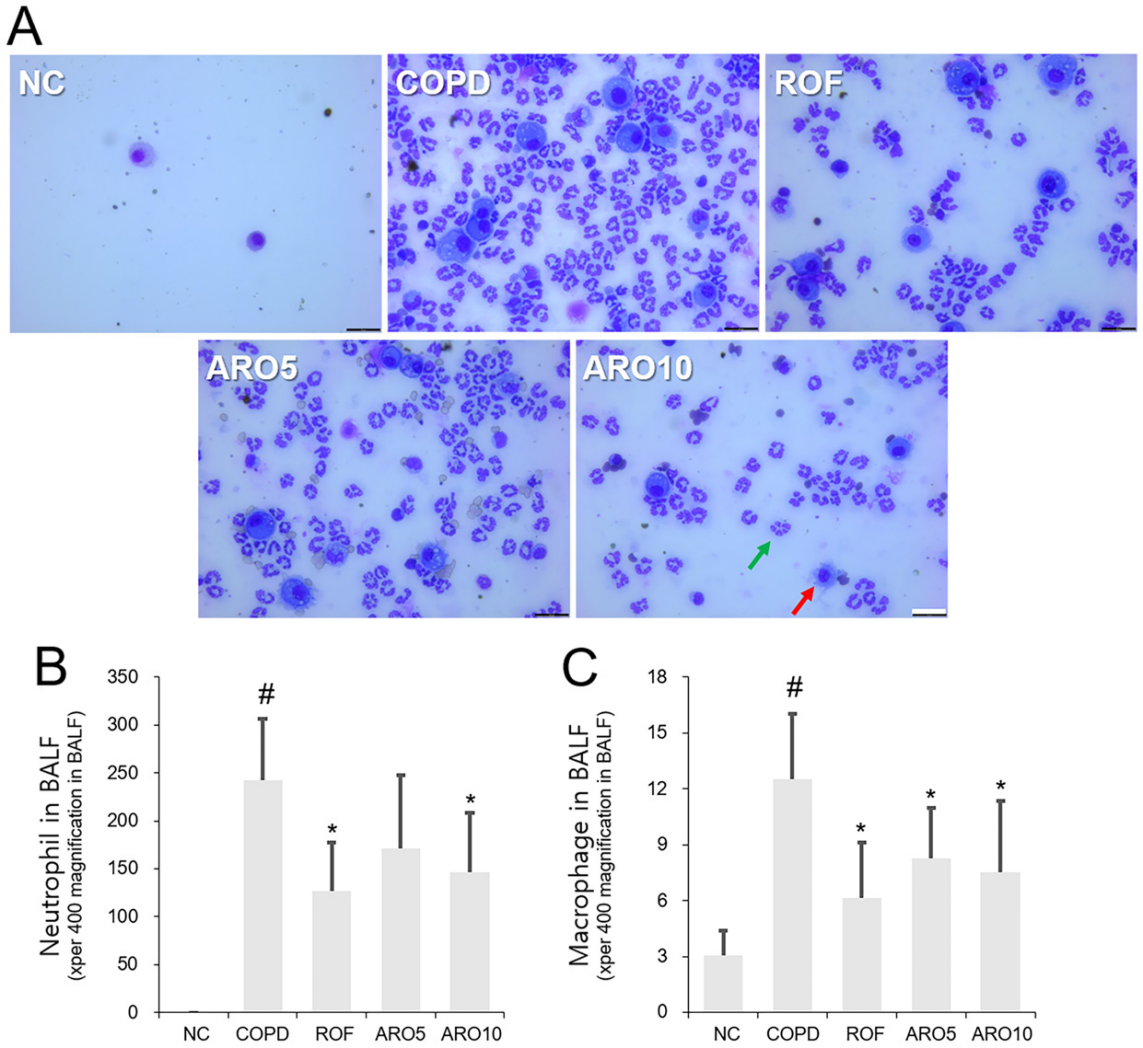
ARO inhibits neutrophils/macrophages influx in experimental COPD mice. (**A**) The images of neutrophils/macrophages were obtained using Diff-Quik staining and microscopy (magnification, x400; scale bar, 25 μm). Number of (**B**) neutrophils and (**C**) macrophages in BAL fluid of mice (green arrows indicate the neutrophils and red arrows indicate the macrophages). Data are expressed as the mean ± SD (^#^*p* < 0.05 for comparison with normal control; *p < 0.05 for comparison with COPD group). NC: normal control mice; COPD: cigarette smoke exposed/LPS-administered mice; ROF: 5 mg/kg ROF-treated COPD mice; ARO 5: 5 mg/kg ARO-treated COPD mice; and ARO 10: 10 mg/kg ARO-treated COPD mice.

Phenolic compounds that inhibit immune cell inflow ameliorate bronchial inflammation in mice with COPD [18]. Therefore, we first examined whether ARO exerts inhibitory effects on neutrophil/macrophage influx. As shown in Fig. 1A and 1B, the influx of these cells was upregulated in the BAL fluid of the COPD group, which was significantly decreased by ARO oral administration (5 and 10 mg/kg). The inhibition rates of ARO on neutrophil counts were 47.8% (5 mg/kg ROF), 29.5% (5 mg/kg ARO), and 39.6% (10 mg/kg ARO). The inhibition rates of ARO on macrophage counts were 51.2% (5 mg/kg ROF), 34.1% (5 mg/kg ARO), and 40.0% (10 mg/kg ARO). The inhibitory effect of ARO (10 mg/kg) on cell influx was comparable to that of 5 mg/kg ROF, which was used as a positive control.

### ARO Decreased the Concentration of Molecules in Mice with COPD

**Fig. 2:**
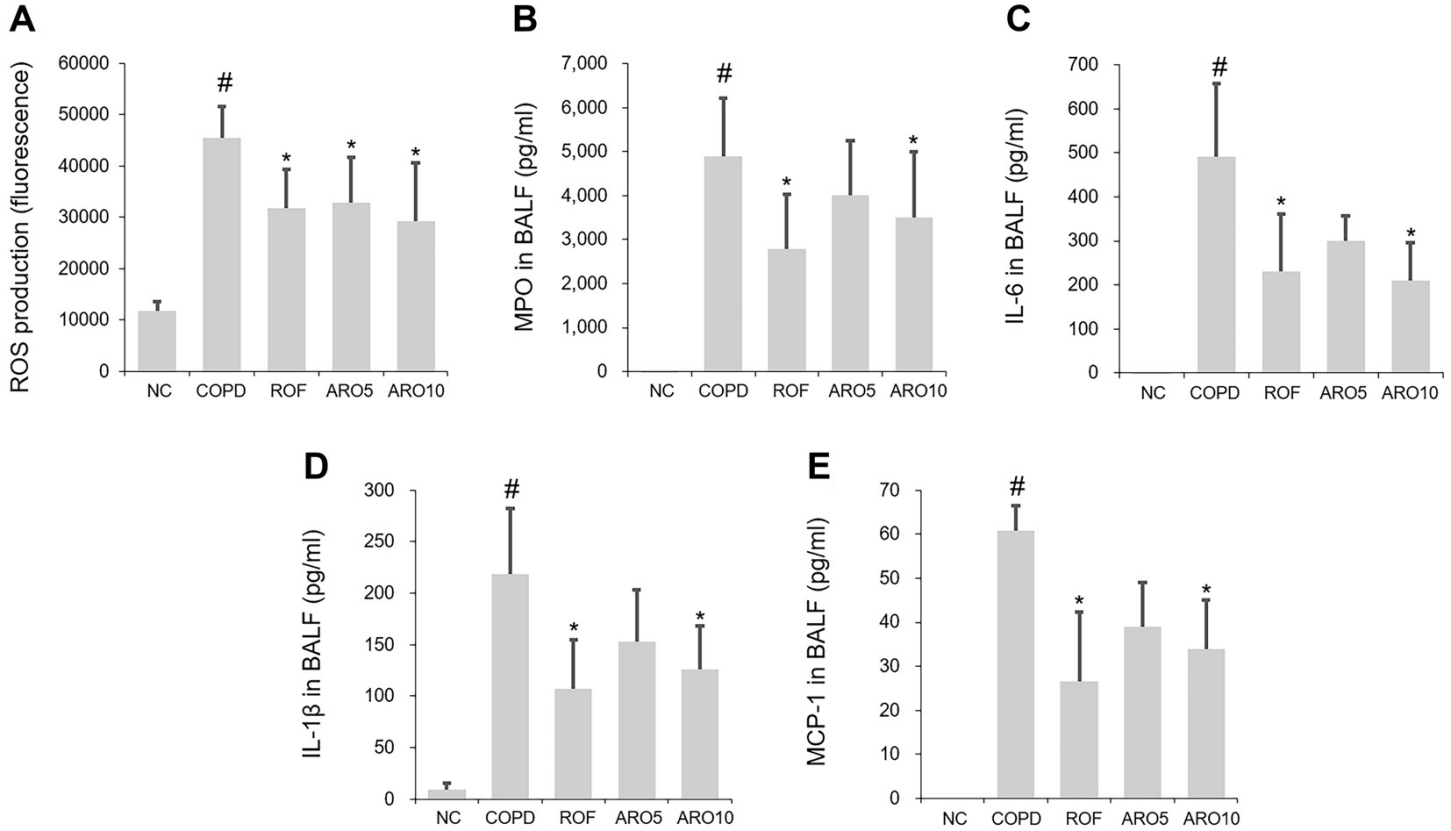
ARO reduces the concentration of inflammatory molecules in experimental COPD mice. (**A**) Total cellular ROS level in BAL fluid cells was determined using 2',7'-dichlorodihydrofluorescein diacetate (DCF-DA). Levels of inflammatory molecules, such as (**B**) MPO (**C**) IL-6, (**D**) IL-1β, and (**D**) MCP-1 in BAL fluid, were detected by ELISA. Data are expressed as the mean ± SD (^#^*p* < 0.05 for comparison with normal control; *p < 0.05 for comparison with COPD group). NC: normal control mice; COPD: cigarette smoke exposed/LPS-administered mice; ROF: 5 mg/kg ROF-treated COPD mice; ARO 5: 5 mg/kg ARO-treated COPD mice; and ARO 10: 10 mg/kg ARO-treated COPD mice.

Increased levels of ROS, MPO, IL-6, IL-1β, and MCP-1 in BAL fluid have been confirmed in COPD mice [11, 18, 19]. In this study, a notable upregulation of these molecules was observed in the BAL fluid of mice with COPD (Fig. 2A-2E). This tendency was mitigated by ARO. The inhibition rates of ARO on ROS production were 30.1%(5 mg/kg ROF), 27.4% (5 mg/kg ARO), and 35.5% (10 mg/kg ARO). The inhibition rates of ARO on MPO production were 42.8% (5 mg/kg ROF), 18.2% (5 mg/kg ARO), and 28.4% (10 mg/kg ARO). The inhibition rates of ARO on IL-6 production were 53.1% (5 mg/kg ROF), 38.8% (5 mg/kg ARO), and 57.4% (10 mg/kg ARO). The inhibition rates of ARO on IL-1β production were 50.9% (5 mg/kg ROF), 29.9% (5 mg/kg ARO), and 42.2%(10 mg/kg ARO). The inhibition rates of ARO on MCP-1 production were 56.3% (5 mg/kg ROF), 35.8% (5 mg/kg ARO), and 44.4% (10 mg/kg ARO). In general, the inhibitory effects of 10 mg/kg ARO on the formation of these molecules were comparable to those of 5 mg/kg ROF.

### Reduced Cell Accumulation and NE Expression by ARO in Mice with COPD

**Fig. 3:**
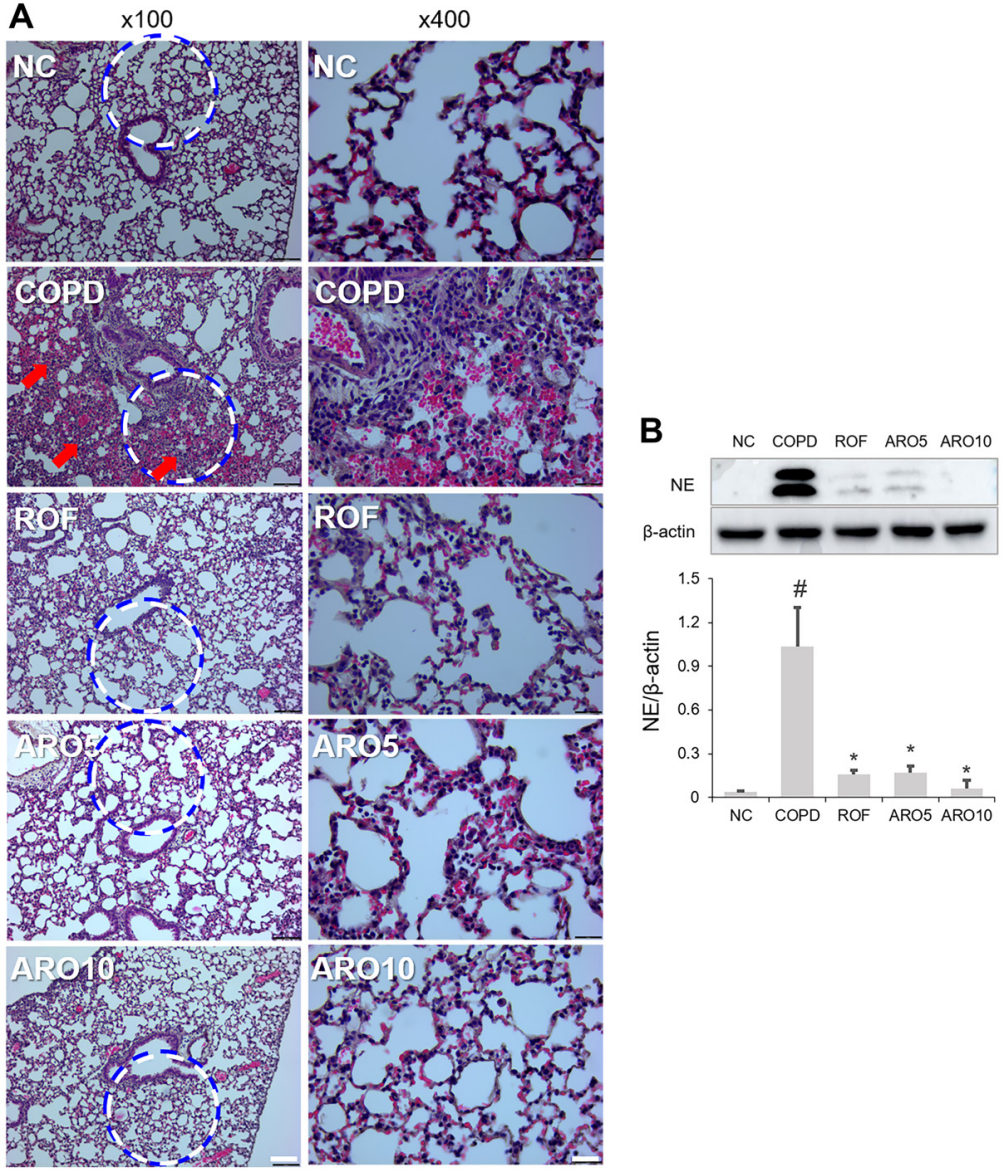
Cell accumulation and elastase expression were reduced by ARO in experimental COPD mice. (**A**) The histological changes in lungs of mice, which show cell accumulation, were assessed using H&E staining (left panel: magnification, 100×; scale bar, 100 μm; right panel: magnification, 400×; scale bar, 25 μm). (**B**) The expression of neutrophil elastase was analyzed using western blotting. Quantitative analysis of neutrophil elastase was performed using ImageJ software. Data are expressed as the mean ± SD (^#^*p* < 0.05 for comparison with normal control; *p < 0.05 for comparison with COPD group). NC: normal control mice; COPD: cigarette smoke exposed/LPS-administered mice; ROF: 5 mg/kg ROF-treated COPD mice; ARO 5: 5 mg/kg ARO-treated COPD mice; and ARO 10: 10 mg/kg ARO-treated COPD mice.

Next, histological changes were observed using H&E staining in the lungs of COPD mice. The results showed that the COPD group had higher cell accumulation compared to the normal control (NC) group (Fig. 3A). However, the ARO-treated group showed reduced cell accumulation compared to the COPD group. NE concentration was increased in an in vivo study of COPD [22]; thus, we measured the inhibitory effect of ARO on NE level using western blotting. NE expression in the lungs of the COPD group was significantly reduced by ARO treatment (Fig. 3B and 3C).

### ARO Reduced Mucus and CREB Activation in Mice with COPD

**Fig. 4:**
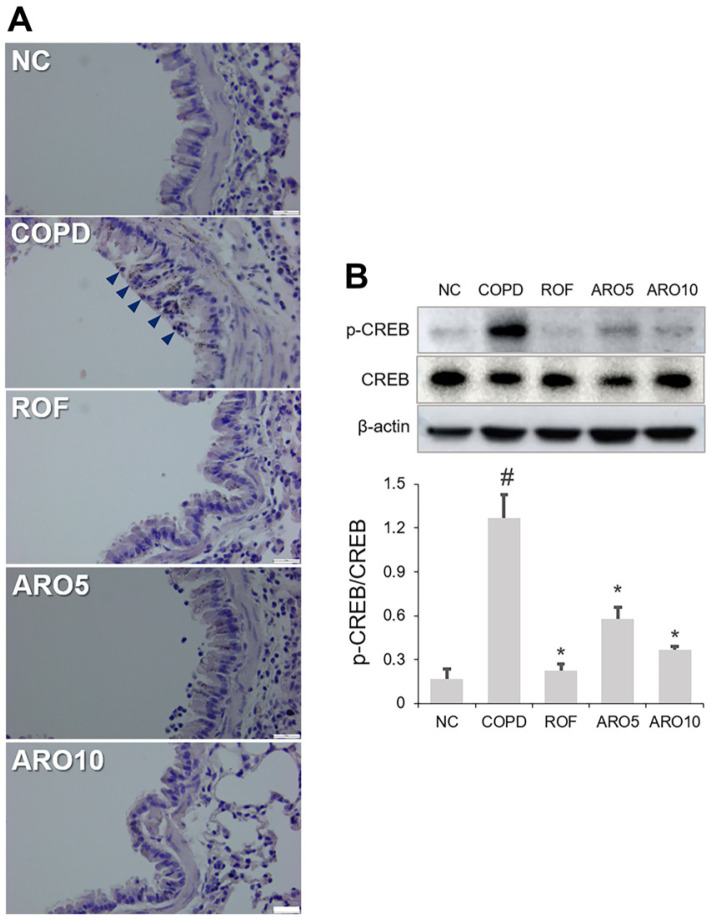
ARO attenuates mucus formation and CREB activation in experimental COPD mice. (**A**) The histological changes in lungs of mice, which indicate mucus formation, were assessed using PAS staining (magnification, 400×; scale bar, 25 μm). (**B**) The activation of CREB was analyzed using western blotting. Quantitative analysis of phosphorylated (p)-CREB was performed using ImageJ software. Data are expressed as the mean ± SD (^#^*p* < 0.05 for comparison with normal control; *p < 0.05 for comparison with COPD group). NC: normal control mice; COPD: cigarette smoke exposed/LPSadministered mice; ROF: 5 mg/kg ROF-treated COPD mice; ARO 5: 5 mg/kg ARO-treated COPD mice; and ARO 10: 10 mg/kg ARO-treated COPD mice.

As shown in Fig. 4A, an increase in mucus formation in the airway epithelium was confirmed in the COPD group using PAS staining, and this increase was restrained in the ARO-treated COPD group. CREB activation is associated with mucus formation [33, 34]. To examine whether ARO could modulate CREB activation, the expression levels of phosphorylated (p)-CREB in the lungs of mice were examined. The results showed that the upregulation of p-CREB in the lungs of the COPD group was notably attenuated by ARO (Fig. 4B and 4C).

### ARO Attenuated MAPK Activation in Mice with COPD

**Fig. 5:**
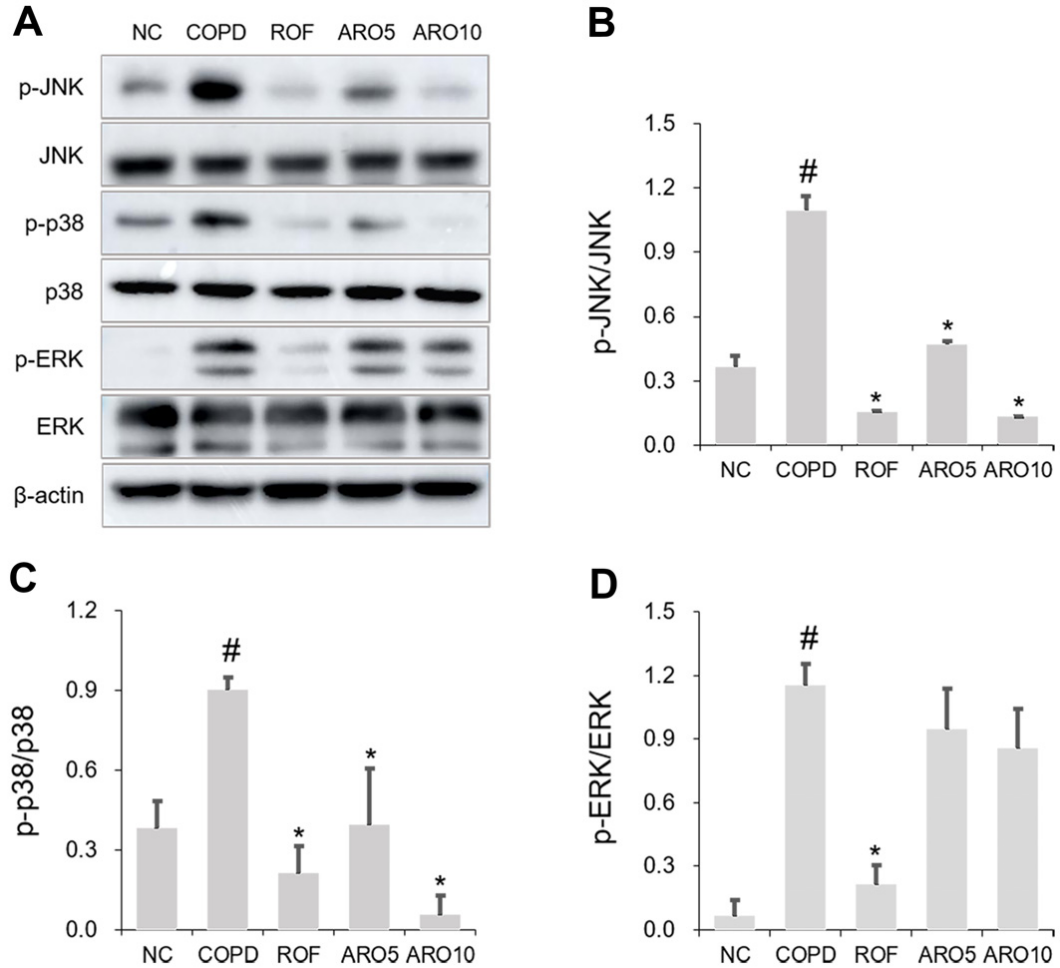
ARO inhibits MAPK activation in experimental COPD mice. (**A**) The activation of JNK, p38, and ERK in lungs of mice was analyzed using western blotting. Quantitative analysis of (**B**) p-JNK, (**C**) p-p38, and (**D**) p-ERK was performed using ImageJ software. Data are expressed as the mean ± SD (^#^*p* < 0.05 for comparison with normal control; *p < 0.05 for comparison with COPD group). NC: normal control mice; COPD: cigarette smoke exposed/LPS-administered mice; ROF: 5 mg/kg ROF-treated COPD mice; ARO 5: 5 mg/kg ARO-treated COPD mice; and ARO 10: 10 mg/kg ARO-treated COPD mice.

As described previously [14-19], activation of JNK, p38, and ERK is an important event in COPD development. Therefore, we explored whether ARO affects MAPK activation in a murine model of COPD. As shown in Fig. 5A-5D, the expressions of p-JNK, p-p38, and p-ERK were significantly increased in the lungs of the COPD group; however, they were reduced in the lungs of the ARO-treated COPD group. Particularly, the inhibitory effect of ARO on JNK and p38 activation was much stronger than that on ERK activation.

### ARO Reduced NF-κB Activation in Mice with COPD

**Fig. 6:**
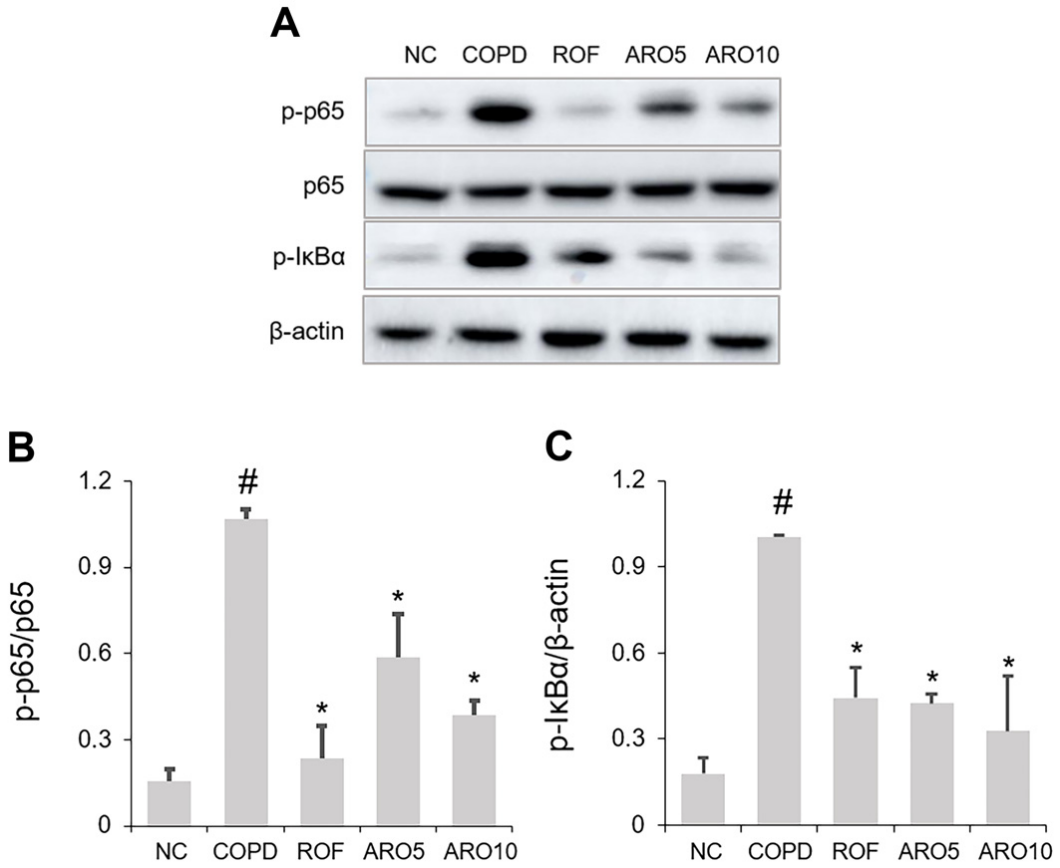
ARO inhibits NF-κB activation in experimental COPD mice. (**A**) The activation of NF-κB p65 and IκBα was analyzed using western blotting. Quantitative analysis of (**B**) p- NF-κB p65 and (**C**) p-IκBα was performed using ImageJ software. Data are expressed as the mean ± SD (^#^*p* < 0.05 for comparison with normal control; *p < 0.05 for comparison with COPD group). NC: normal control mice; COPD: cigarette smoke exposed/LPS-administered mice; ROF: 5 mg/kg ROF-treated COPD mice; ARO 5: 5 mg/kg ARO-treated COPD mice; and ARO 10: 10 mg/kg ARO-treated COPD mice.

NF-κB inactivation alleviates bronchial inflammation in COPD mice [20], and we therefore investigated the inhibitory effects of ARO on NF-κB activation in this study. As shown in Fig. 6A-6C, NF-κB p65 and IκBα were activated in the lungs of COPD mice; this activation was decreased by ARO administration.

### ARO Diminished NLRP3 Activation in Mice with COPD

**Fig. 7:**
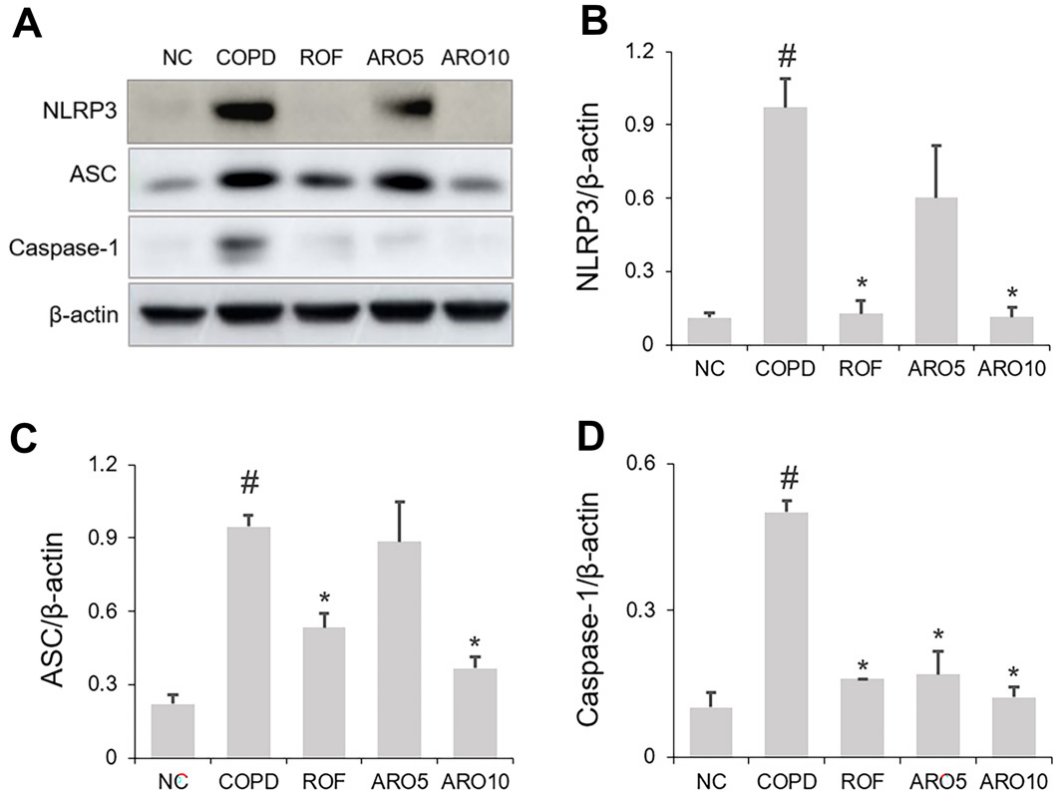
ARO inhibits NLRP3 activation in experimental COPD mice. (**A**) The expression of NLRP3, ASC, and caspase-1 was analyzed using western blotting. Quantitative analysis of (**B**) NLRP3, (**C**) ASC, and (**D**) caspase-1 was performed using ImageJ software. Data are expressed as the mean ± SD (^#^*p* < 0.05 for comparison with normal control; *p < 0.05 for comparison with COPD group). NC: normal control mice; COPD: cigarette smoke exposed/LPS-administered mice; ROF: 5 mg/kg ROF-treated COPD mice; ARO 5: 5 mg/kg ARO-treated COPD mice; and ARO 10: 10 mg/kg ARO-treated COPD mice.

Next, we investigated the effects of ARO on NLRP3 inflammasome activation. As shown in Fig. 7A and 7B, the expression of NLRP3 in the lungs of the COPD mice was increased; however, this increase was suppressed by the oral administration of ARO. Furthermore, the increase in ASC and Caspase-1 expression in the COPD group was suppressed by ARO treatment (Fig. 7A, 7C, and 7D).

## Discussion

Neutrophil- and macrophage-derived molecules, such as toxic molecules, cytokines, and chemokines, promote airway inflammation and lung damage in COPD. Thus, controlling the inflow of neutrophils/macrophages and the formation of these cell-mediated molecules are important for ameliorating bronchial inflammation and lung damage. In the present study, ARO excellently inhibited the influx of neutrophils/macrophages and the formation of ROS/MPO/NE/IL-6/IL-1β/MCP-1 in COPD mice. This ability was comparable to that of the positive control ROF. These observations indicated that ARO may exert anti-inflammatory effects on bronchial inflammation in COPD.

As unnecessary mucus generation impedes airflow and CREB activation is closely associated with mucus formation [33, 34], controlling CREB activation may improve airway flow restriction. ARO inhibited both mucus formation and CREB activation in experimental mice with COPD, indicating that ARO exerts a modulatory effect on mucus formation.

Previously, p38 was shown to be activated in the alveolar spaces of COPD patients [35]. Earlier clinical research demonstrated that NF-κB expression was upregulated in the bronchial epithelium of COPD patients [36]. Meanwhile, a recent clinical study confirmed a significant increase in NLRP3/caspase-1/IL-1β expression in COPD patients [37]. A preclinical study also showed that suppression of JNK/p38/ERK MAPK ameliorates inflammatory responses in a murine model of COPD [22]. Previous and current observations have indicated the usefulness of NF-κB inactivation in ameliorating airway inflammation in mice with COPD [11, 38]. Furthermore, inhibition of the NLRP3 inflammasome disrupts the expression of IL-1β, a therapeutic target for COPD treatment [21]. These observations have demonstrated that targeting MAPK/NF-κB/NLRP3 inflammasome pathways may be an effective therapeutic approach against COPD. In the present study, the inhibitory effects of ARO on MAPK (JNK and p38)/NF-κB/NLRP3 activation were notable, and its ability was comparable to that of ROF. These results highlight the potential of ARO as an adjuvant for COPD.[Fig F8]

Natural products and their bioactive compounds exhibit various biological activities, including anti-inflammatory and antioxidant effects [17, 39]. Accumulating studies have proven that bioactive compounds ameliorate lung inflammation in COPD mice by inducing MAPK/NF-κB/NLRP3 inactivation. Recently, ARO was shown to exert anti-asthmatic effects by suppressing NF-κB activation [30]. In addition, Ma *et al*. showed that ARO ameliorated endotoxin-induced kidney inflammation by attenuating MAPK/NF-κB activation [40]. In the present study, ARO had a regulatory effect on both MAPK/NF-κB and NLRP3 activation in COPD. Therefore, ARO may alleviate bronchial inflammation in patients with COPD.

Collectively, our results demonstrate that ARO ameliorated bronchial inflammation by suppressing immune cell inflow, inflammatory molecule formation, and mucus formation. These effects were accompanied with suppressive effect on MAPK/NF-κB/NLRP3 activation. Overall, ARO showed excellent efficacy at a dose of 10 mg/kg, highlighting its potential for the development of anti-COPD adjuvants.

## Figures and Tables

**Fig. 8 F8:**
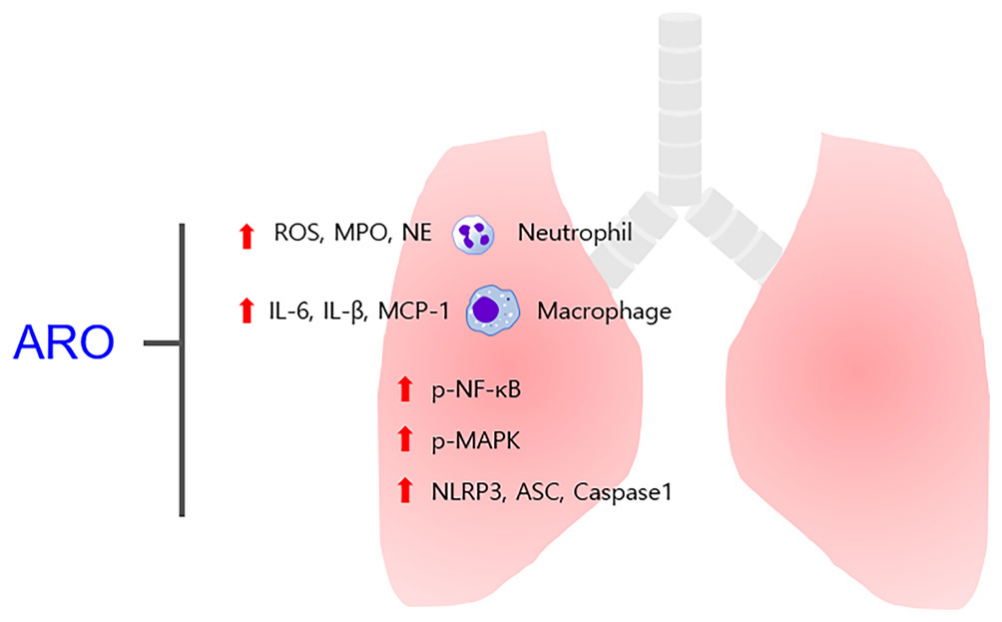
Ameliorative effects of ARO on bronchial inflammation in experimental COPD mice. Oral administration of ARO reduces neutrophil/macrophage inflow and ROS/MPO/elastase/IL-6/IL-1β/MCP-1 formation in COPD mice. These effects of ARO are accompanied by its modulatory effect on MAPK/NF-κB/NLRP3 activation.

**Table 1:** List of antibodies.

NO	Primary antibody	Company	Molecular weight	Host	Secondary antibody
1	NE (bs-6982R)	Bioss Antibodies	26	Rabbit	Goat anti rabbit-HRP
2	p-CREB (9198s)	Cell Signaling	43	Rabbit	Goat anti rabbit -HRP
3	CREB (9197s)	Cell Signaling	43	Rabbit	Goat anti rabbit -HRP
4	p-JNK (4668S)	Cell Signaling	46, 54	Rabbit	Goat anti rabbit -HRP
5	JNK (9252S)	Cell Signaling	46, 54	Rabbit	Goat anti rabbit -HRP
6	p-p38 (sc7973)	Santa Cruz	38	Mouse	Goat anti mouse-HRP
7	p38 (sc-7972)	Santa Cruz	38	Mouse	Goat anti mouse-HRP
8	p-ERK (9101s)	Cell Signaling	42, 44	Rabbit	Goat anti rabbit-HRP
9	ERK (9102s)	Cell Signaling	42, 44	Rabbit	Goat anti rabbit-HRP
10	p-p65 (3033S)	Cell Signaling	65	Rabbit	Goat anti rabbit-HRP
11	p65 (sc-8008)	Santa Cruz	65	Mouse	Goat anti mouse-HRP
12	p-IκBα (2859S)	Cell Signaling	40	Rabbit	Goat anti rabbit-HRP
13	NLRP3 (15101s)	Cell Signaling	110	Rabbit	Goat anti rabbit-HRP
14	ASC (sc-514414)	Santa Cruz	24	Mouse	Goat anti Mouse-HRP
15	Caspase-1 (sc56036)	Santa Cruz	45	Mouse	Goat anti Mouse-HRP
16	β-action (sc-47778)	Santa Cruz	43	Mouse	Goat anti mouse-HRP
